# Structural quality criteria of emergency departments in Vienna

**DOI:** 10.1007/s00508-025-02541-7

**Published:** 2025-05-19

**Authors:** Harald Herkner, Alexandra J. Lipa, Philip Eisenburger, Wilhelm Behringer, Alexander Spiel, Sonja Mahrer, Moritz Haugk, Rainer Thell, Marie-Kathrin Breyer, Nicole Biber, Edith Doberer, Anna Kreil, Michael Schwameis

**Affiliations:** 1 Department of Emergency Medicine, Medical University of Vienna, Währinger Gürtel 18–20, 1090 Vienna, Austria; 2 Department of Emergency Medicine and Internal Medicine, Klinik Floridsdorf, Vienna Healthcare Group, Vienna, Austria; 3 Department of Emergency Medicine, Klinik Ottakring, Vienna Healthcare Group, Vienna, Austria; 4 Emergency Department, Klinik Favoriten, Vienna Healthcare Group, Vienna, Austria; 5 Department of Emergency Medicine, Klinik Hietzing, Vienna Healthcare Group, Vienna, Austria; 6 Department of Internal Medicine 2, Emergency Department, Klinik Donaustadt, Vienna Healthcare Group, Vienna, Austria; 7 Department of Respiratory and Pulmonary Diseases, Klinik Penzing, Vienna Healthcare Group, Vienna, Austria; 8 Emergency Department, Department of Internal Medicine I, Hanusch Krankenhaus, Vienna, Austria; 9 Emergency Department, Barmherzige Brüder Hospital Vienna, Vienna, Austria; 10 Emergency Department, Klinik Landstraße, Vienna Healthcare Group, Vienna, Austria

**Keywords:** Emergency medicine, Tertiary healthcare, Quality indicators, Hospitals, Austria

## Abstract

**Background:**

In 2020, the Austrian Association of Emergency Medicine proposed structural quality criteria for in-hospital emergency care in Austria. However, it has not yet been assessed how these criteria apply to existing emergency departments.

**Methods:**

All in-hospital emergency departments across Vienna were surveyed using a structured assessment based on published proposed structural quality criteria. A total of 54 criteria were analysed, each rated on a scale of 3 (comprehensive care), 2 (standard care), 1 (basic care), or 0 (not met).

**Results:**

Among 16 hospitals, we identified 10 emergency departments. The scores ranged from 87 points (54%) to 151 points (95%). None of the departments met all structural quality criteria. Overall, across all emergency departments, 69% of the criteria aligned with comprehensive care, 7% with standard care and 3% with basic care, while 21% of the criteria were not met at all.

**Conclusions:**

A set of proposed structural quality criteria for emergency departments could be quantitatively assessed. While the published criteria and the observed infrastructure are largely consistent, there is significant potential for improvement in both the definition of the criteria and the criteria per se. The extent to which these structural quality criteria are useful for assessing the classification of tiered care models requires further studies in different regions.

## Background

In-hospital emergency medicine is globally a well-established healthcare structure, serving as a critical interface for acute medical cases across prehospital, outpatient, and inpatient care. However, in German-speaking countries, emergency departments have become established area-wide only in recent years. Noteworthy, Austria has an acknowledged specialization for in-hospital acute and emergency medicine since 2025.

In Austria, structural quality criteria are outlined in the Austrian Health Care Structural Plan (“Österreichischer Strukturplan Gesundheit”, ÖSG) [1] to ensure a standardized, high-quality healthcare system across outpatient and inpatient sectors. In addition to process- and outcome-related quality, structure-related quality is an integral part of quality management in the Austrian healthcare system and is enshrined in the Healthcare Quality Act [2]. By definition (Sect. 2 GQG), structure-related quality is understood as the “sum of material and personnel resources in quantitative and qualitative terms”. Currently, the ÖSG specifies Outpatient First Aid Centers (“Zentrale Ambulante Erstversorgung”, ZAE) for the initial treatment of acute cases in acute care hospitals at the level of general medicine. This includes the initial care of patients without an appointment (unplanned patient contacts) with acute symptoms; assessment of urgency according to standardized methodology (triage); appropriate assessment and, if necessary, treatment and/or referral, also to the outpatient sector; if necessary, observation for up to a maximum of 24 h in outpatient hospital treatment. For a ZAE, structural quality criteria are already defined in the ÖSG (ÖSG 2023—3.2.4). Moreover, the ÖSG states that a ZAE may be linked to an emergency department (“zentrale Notaufnahme”, ZNA), but there is no definition of ZNA in this document at all. For instance, it does not account for the resources required to treat acute critically ill patients in emergency departments, despite their role as holistic acute care providers. Specifying structural quality criteria for all levels of emergency facilities is therefore essential, including those for emergency departments (ZNA).

To address this gap, the Austrian Association of Emergency Medicine (AAEM; www.aaem.at) proposed a detailed framework for tiered emergency care—including comprehensive, standard, and basic care—ensuring coverage of all emergency patients, including critically ill cases in 2020 [3].

Germany legally defined structural quality criteria for in-hospital emergency medicine in 2018 [4], while Switzerland has comprehensive standards for prehospital care but none for in-hospital settings [5]. Beyond structural quality criteria for grading of emergency care, other classification models exist, such as Denmark’s description of emergency facility organizational forms [6].

This study aims to systematically assess the representation of the proposed structural quality criteria across Vienna’s hospital emergency departments.

## Methods

This study included all public acute-care hospitals in Vienna with general or multispecialty services (http://www.sozialministerium.at/Themen/Gesundheitssystem/Krankenanstalten/Krankenanstalten-und-selbststaendige-Ambulatorien-in-Oesterreich/Krankenanstalten-in-Oesterreich.html, Table 1). We defined emergency department as any in-house facility, which has the core task to handle medical emergencies and acute illnesses (e.g., central emergency outpatient department, emergency room, department for emergency medicine, ZNA, ZAE). We identified all hospitals which run an emergency department, and subsequently invited these to participate in our study. Hospitals were excluded if they did not run a dedicated emergency department. The study was conducted between July 2023 and June 2024; the tabulated information provided is up-to-date as of October 2024.

**Table 1:** Public acute-care hospitals in Vienna

Hospital	Hospital type	Hospital beds *n* =	Intensive care in the hospital	Facilities in the hospital	Name of the emergency department
Universitätsklinikum AKH Wien	Central hospital	1440	AN, CH, GEM, HCH, IM, KIJU, KJC, NCH, NEU, PCH, PSY	COR, CT, SPECT, MRI, PET, STR	Universitätsklinik für Notfallmedizin
Krankenhaus der Barmherzigen Brüder	Specialist hospital	383	AN, IM	CT, SPECT, MRI	Zentrale Aufnahme und Erstversorgung (ZAE)
Krankenhaus der Barmherzigen Schwestern	Standard hospital	195	AN, IM	CT	–
Evangelisches Krankenhaus	Standard hospital	226	GEM	CT, SPECT, MRI	–
Klinik Favoriten	Specialist hospital	629	AN, IM, KIJU	COR, CT, MRI, STR	Internistische Notaufnahme
Hanusch-Krankenhaus	Specialist hospital	320	AN	COR, CT, SPECT, MRI, PET	Zentrale Notaufnahme
Herz-Jesu Krankenhaus	Standard hospital	162	AN	CT	–
Franziskus Spital	Standard hospital	240	GEM	CT	–
Klinik Hietzing	Specialist hospital	759	AN, CH, IM, NEU	CT, SPECT, MRI, STR	7. Medizinische Abteilung – Innere Medizin mit Notfallmedizin
Klinik Landstraße	Specialist hospital	600	AN, IM, KIJU	COR, CT, SPECT, MRI, PET	Zentrale Notaufnahme – Aufnahmeabteilung und Erstuntersuchungs-ambulanz
St. Josef Krankenhaus Wien	Standard hospital	175	GEM, KIJU	CT	–
Klinik Ottakring	Specialist hospital	750	AN, IM, KIJU	COR, CT, SPECT, MRI, PET, STR	Zentrale Notaufnahme – Abteilung für Notfallmedizin und Innere Medizin (ZNA)
Göttlicher Heiland Krankenhaus	Standard hospital	257	AN, IM	COR, CT, MRI	–
Klinik Donaustadt	Specialist hospital	842	AN, CH, IM, KIJU, ORTR	COR, CT, SPECT, MRI, PET, STR	Notfallambulanz mit Infektionsambulanz
Klinik Floridsdorf	Specialist hospital	646	AN, IM, KIJU, PUL	COR, CT, MRI	Abteilung für Notfallmedizin und Innere Medizin (ZNA)
Klinik Penzing	Specialist hospital	341	IM, PUL	CT, MRI	Notfallambulanz

Hospital Care Levels defined according to KAKuG, Krankenanstalten- und Kuranstaltengesetz (Federal Act on Hospitals and Sanatoria); Intensive care: *AN *anaesthesiology; *IM* internal medicine; *GEM *mixed; *KIJU *children and adolescents; *KJC* paediatric surgery; *HCH* cardiac surgery; NCH neurosurgery; *NEU* neurology; *CH* surgery; *ORTR* orthopaedics and trauma surgery; *PUL* pneumology; *PCH* plastic surgery; *PSY* psychiatry. Facilities in the hospital: *COR* coronary angiography; *CT* computed tomography; *MRI* magnetic resonance imaging, *PET* positron emission tomography; *SPECT* single-photon emission computed tomography; *SRT* radiation therapy

Based on the proposed structural quality criteria developed by the AAEM [3], a list of 54 criteria was sent to the department heads of each emergency department. The proposed criteria include organizational aspects (e.g. personnel) and structural aspects (e.g. beds, availability of imaging). The investigators (H.H, A.J.L, and M.S) adapted this set of criteria in order to gather data in a feasible manner. In a pilot phase, the table was reviewed by the head of one department. Among the proposed published criteria, the following were omitted from the survey in agreement: haemodialysis, paediatric first aid, intervention room, labelling for disaster operations, room class suitable for interventions or induction of anaesthesia, intensive care monitor in the acute care area, ICP monitoring, transcranial Doppler ultrasound, EEG, continuous EEG monitoring. An electronic spreadsheet was then sent to all department heads. In case of uncertainties, the respondents contacted the study team directly. Each criterion was scored according to care level: comprehensive care (3 points), standard care (2 points), basic care (1 point), or not fulfilled (0 points). As published, comprehensive care should be available if the annual case load exceeds 20,000, standard care for 20,000–10,000, and basic care for emergency departments with less than 10,000 patients per year. Criteria, for which multiple care levels applied were assigned the highest number of points (e.g. magnetic resonance imaging availability in the same hospital = 3 points). The maximum possible score was 162 points (54 criteria × 3 points). Any ambiguities in scoring were clarified through discussions with the study team considering the original publication [3]. Results were pseudonymized, with hospitals represented by letters. We presented data as absolute numbers and relative frequencies. As this was not a random sample in the frequentist sense, we did not estimate random variability or perform hypothesis tests.

## Results

A total of 16 public acute-care hospitals were identified in Vienna. Table 1 lists these hospitals, including the hospital type [7] and other organisational attributes. Among these, 10 hospitals operate an in-hospital emergency department, all of which responded and were included in the analysis.

The 54 structural quality criteria are detailed in Fig. 1. Structural quality criteria scores ranged from 87 (54%) to 151 (95%) points. Across all emergency departments, 376 (70.9%) criteria aligned with comprehensive care standards, 39 (7.2%) with standard care, and 16 (3.0%) with basic care, while for 109 (20.6%) instances none were met. The distribution of structural quality criteria by care level for each emergency facility is illustrated in Fig. 2.

**Fig. 1:**
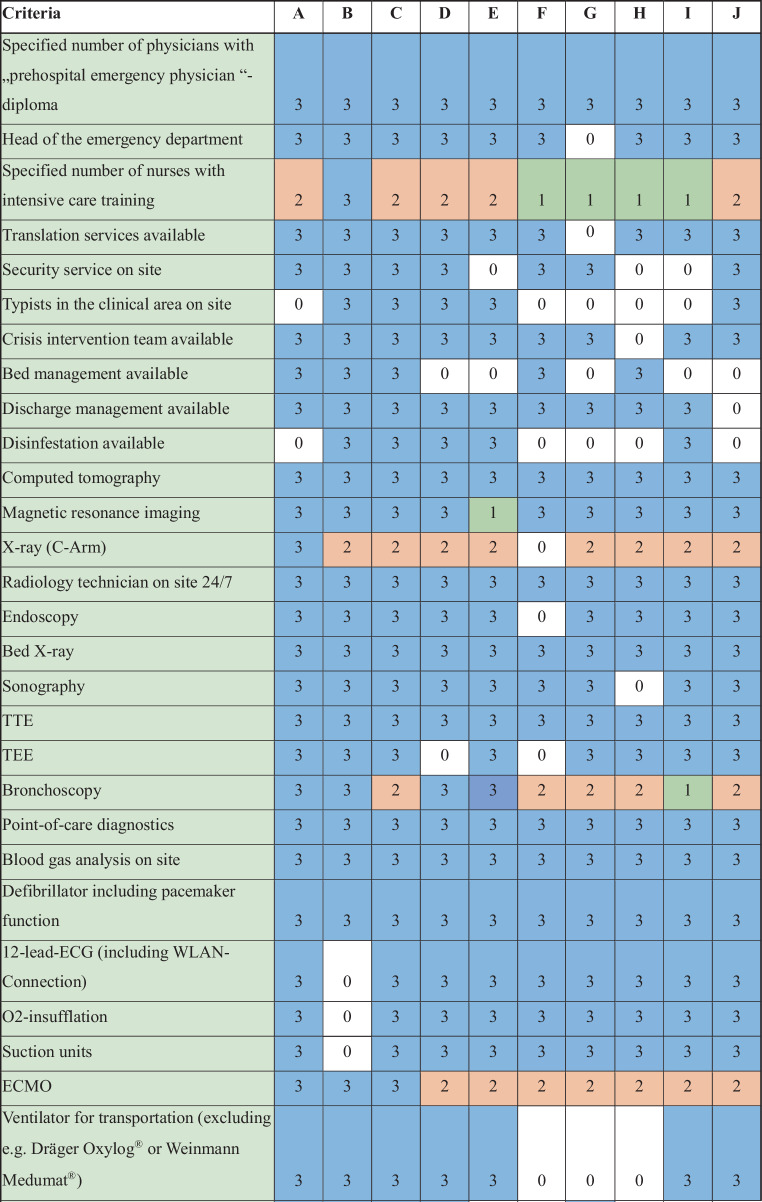
List of 54 proposed structural quality criteria for emergency departments in Austria. *TEE* transoesophageal echocardiography; *TTE* transthoracic echocardiography. 0 (white) = criteria not met; 1 (green) = basic care; 2 (orange) = standard care; 3 (blue) = comprehensive care. A–J: included hospitals. *ECG* electrocardiogram, *ECMO* extracorporal membrane oxygenator

**Fig. 1:**
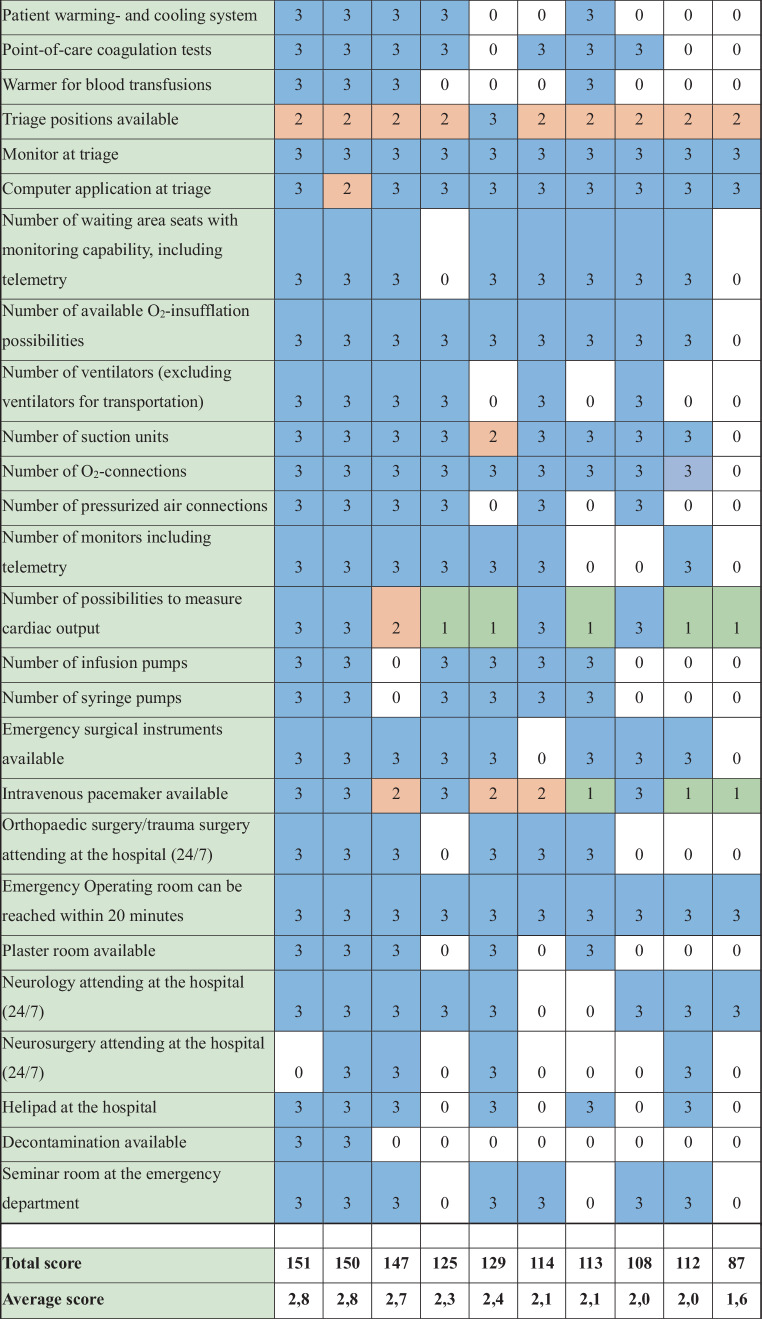
(continued)

**Fig. 2:**
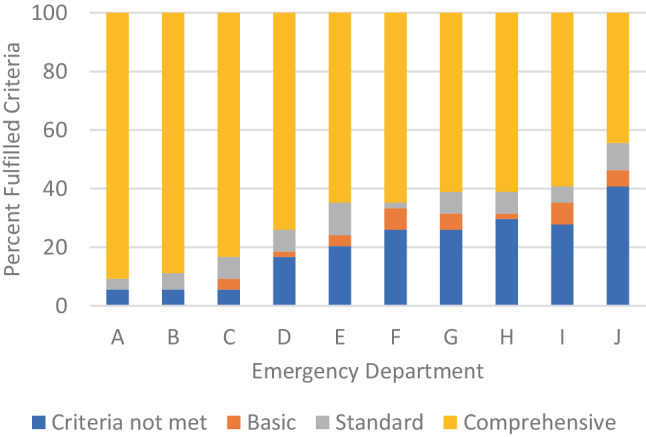
Distribution of structural quality criteria ratings across emergency departments. The emergency departments are labelled A‑J on the x‑axis. The y‑axis shows the relative number of fulfilled criteria (%). The colour code indicates the highest fulfilled category for each department

No emergency department met all structural quality criteria. Two departments achieved at least 90% of the criteria for comprehensive care. Eleven criteria were consistently met across all departments—10 corresponding to comprehensive care and one to standard care standards. Additionally, 12 criteria aligned with comprehensive care level in 9 out of 10 departments. A total of 32 criteria showed variability in care levels among emergency departments. For the following 4 criteria, all levels of care were represented: 1) number of registered nurses with intensive care training, 2) availability of bronchoscopy, 3) availability of a transvenous pacemaker, 4) availability of a C-arm (X-ray). The criterion “extracorporeal membrane oxygenation (ECMO)” was met only by the three best-equipped emergency departments.

## Discussion

None of the emergency departments in Vienna fully met the proposed structural quality criteria. However, two departments met at least 90% of the criteria at a comprehensive care level. The majority of emergency departments scored at least 2 points per criterion on average, classifying them as standard care providers.

The structural quality criteria reflect expert consensus, serving as an experience-based recommendation for the essential infrastructure of emergency departments. Although a high overall compliance rate was observed, 21% of the criteria were not met even at the basic care level. These deficiencies primarily concerned nonmedical specialist staff, intensive care equipment, specialist availability as well as hospital-wide support structures and organizational facilities.

This observation aligns with known structural deficits, some of which are also part of a continuous improvement process of the individual emergency departments and hospitals. Interestingly, the criterion ‘triage’ is at the comprehensive care level in one emergency department only, and standard for all others. By the published definition, one triage-position per 20,000 patient contacts per year would be classified as a comprehensive care provider. The threshold of 20,000 patient contacts is also referenced in a recommendation by the German Society for Interdisciplinary Emergency and Acute Medicine (DGINA) and the German Interdisciplinary Association for Intensive Care and Emergency Medicine (DIVI) regarding a corresponding Federal Joint Committee (“Gemeinsamer Bundesauschuss”, GB-A) decision in Germany [8]. Moreover, other criteria are probably overly strict, too. For instance, basic care can be warranted without the possibility of decontamination in every hospital. Similarly, requiring the availability of a neurosurgical departments in the same hospital appears beyond reach for a basic emergency care. Any future revision of the structural quality criteria should ensure that all emergency departments are able to meet at least the baseline requirements.

We found that only a limited number of criteria contributed to the classification of the different care levels of the emergency departments. This is partly due to the nature of the classification system itself, as some criteria represent general minimum requirements (e.g. availability of a defibrillator) that apply equally across all levels. However, significant variations exist in staffing levels, the number of acute care beds, specialized intensive care equipment (e.g. ECMO), and the overall resources available at each hospital. Accordingly, these criteria reflect a concept of tiered emergency care ranging from outpatient level care up to critical care in the emergency department [9], aiming for seamless and timely care models. This also raises the question of whether the focus should be on differentiating between the levels of care or on good equipment for all levels.

In Germany, a tiered system for emergency structures in hospitals was adopted for the first time in 2018 [4]. The objective of this regulation by the G‑BA was the concrete definition of multilevel emergency care, taking into account nonparticipation in emergency care. The G‑BA defined five categories: “type and number of specialist departments”, “number and qualification of the specialist staff to be provided”, “capacity for the care of intensive care patients”, “medical–technical equipment”, “emergency department structures and processes”. Emergency structures of the respective categories were defined and graded so that three levels can be defined for hospitals participating in emergency care: basic emergency care (level 1), advanced emergency care (level 2), and comprehensive emergency care (level 3). If at least the “level 1” criteria are not met or the hospital does not participate in the emergency care system, corresponding discounts must be applied to the remuneration for all services provided by the hospital [4, 10].

In Switzerland, the Swiss Society for Emergency and Rescue Medicine (“Schweizerische Gesellschaft für Notfall-und Rettungsmedizin”, SNGOR) has already provided quality strategies in recent years that cover both prehospital and in-hospital emergency medicine [5]. Despite comprehensively defined structural quality criteria for the prehospital sector [11], Switzerland has yet to implement comprehensive legislative regulations for in-hospital emergency medicine. Moreover, emergency medicine and the establishment of structural quality criteria are not included in the country’s Health 2030 Strategy plan [12].

A limitation of this study is its exclusive focus on a metropolitan setting. Some of the hospitals analysed operate across multiple levels of care simultaneously: in this urban area, there is no strict tiered model of care levels. The study should therefore be carried out in nonmetropolitan areas, as more variability can be expected here as a result of graduated emergency care. However, we are also aware that organizational properties within a country are determined by factors at several levels, such as geospatial, reimbursement, health organization, etc. and may therefore hardly be generalisable globally.

## Conclusion

A set of proposed structural quality criteria for emergency departments could be quantitatively assessed. While the published criteria and the observed infrastructure are largely consistent, there is significant potential for improvement in both the definition of the criteria and the criteria per se. The extent to which these structural quality criteria are useful for assessing the classification of tiered care models requires further studies in different regions.
